# Distinguishing common respiratory pathogens using machine learning of symptom profiles to prioritize diagnostic testing

**DOI:** 10.1371/journal.pdig.0001656

**Published:** 2026-08-21

**Authors:** Chunyan Xiang, Tingting You, Fei Zhou, Dong Liu, Yimin Wang, Bin Cao, Yeming Wang

**Affiliations:** 1 Department of Pulmonary and Critical Care Medicine, Capital Medical University, National Center for Respiratory Medicine, State Key Laboratory of Respiratory Health and Multimorbidity, National Clinical Research Center for Respiratory Diseases, Institute of Respiratory Medicine, Chinese Academy of Medical Sciences, Center of Respiratory Medicine, China-Japan Friendship Hospital, Beijing, China; 2 Scientific Research Center, China-Japan Friendship Hospital, Beijing, China; 3 Tsinghua University-Peking University Joint Center for Life Sciences, Beijing, China; Instituto Politécnico Nacional Escuela Superior de Medicina: Instituto Politecnico Nacional Escuela Superior de Medicina, MEXICO

## Abstract

Clinical symptoms are critical for diagnosing and managing upper respiratory tract infections, yet systematic comparisons across common pathogens remain limited. We aimed to characterize symptom signatures across common respiratory pathogens and to develop approaches that support the prioritization of diagnostic testing across pathogens. Using a large-scale, home-based multiplex PCR testing dataset, we characterized symptom profiles across common respiratory pathogens stratified by age and sex. Hierarchical clustering was applied to group pathogens into symptom-based categories, and a multilayer perceptron (MLP) model was trained to predict these cluster-level categories. A post-hoc refinement combined MLP outputs with contemporaneous epidemiological data to further prioritize likely pathogens within predicted categories. Model performance was evaluated using the area under the receiver operating characteristic curve (AUC), classification accuracy, and related metrics. Symptom profiles overlapped substantially across pathogens, with differences being more categorical than pathogen-specific: RSV, HCoV, HPIV, and HRV were predominantly characterized by respiratory symptoms, whereas influenza A/B and SARS-CoV-2 showed more pronounced systemic manifestations. The MLP model achieved high AUCs (0.80–0.92, depending on age group) with specificities exceeding 75% but lower sensitivities, reflecting a conservative prediction pattern. Integrating cluster-level predictions with contemporaneous epidemiology improved the prioritization of prevalent pathogens, though performance remained limited for less common ones. When expanding predictions to the top three likely pathogens, the model enabled diagnostic test prioritization with an overall accuracy of 0.83 (95% CI, 0.82–0.84). Our study supports the use of readily available symptom data to inform the prioritization of diagnostic testing, particularly during outbreaks or in resource-limited settings where timely laboratory testing is constrained. In addition, the systematic symptom signatures identified across common respiratory pathogens may inform the development and optimization of symptom-based patient-reported outcomes for future antiviral trials.

## Author summary

Respiratory symptoms such as cough, fever, and sore throat are often the first signals that prompt diagnostic testing for respiratory infections. However, many common respiratory pathogens cause highly overlapping symptoms, making it difficult to determine which tests are most relevant based on symptoms alone. In this study, we analyzed a large home-based multiplex testing dataset to systematically compare symptom profiles across nine common respiratory pathogens. We found that while individual symptoms were rarely specific to a single pathogen, symptom patterns could be grouped into broader categories, which were observed across different age and sex groups. By combining these symptom-based categories with machine learning and contemporaneous epidemiological information, we developed an approach to prioritize likely pathogens and narrow the scope of diagnostic testing. Our approach is intended to support early-stage decision-making by helping clinicians and health systems focus diagnostic resources on the most plausible targets. This may be particularly valuable during outbreaks or in resource-limited settings where laboratory capacity is constrained. In addition, the systematic symptom patterns identified in this study provide a structured basis for the future development and optimization of symptom-based patient-reported outcomes in studies of respiratory infections and antiviral interventions.

## Introduction

Upper respiratory infections (URTIs) are among the most common diseases in primary medical care. Globally, the incidence rate of URTIs was 162,484.8 per 100,000 population in 2021 [1]. Although URTIs are usually self-limited and rarely fatal, the symptoms can significantly impair life quality and productivity, causing about 6.4 million DALYs in 2019 globally [2]. The imperative for early and accurate pathogen testing, or the timely initiation of empirical therapy, is to expedite appropriate antiviral treatment while avoiding unnecessary antibiotics, thereby optimizing clinical outcomes for vulnerable populations such as children and the elderly.

Although diagnostic testing methods have improved considerably in recent years, each approach carries distinct advantages and limitations. Nucleic acid amplification tests (NAATs), most notably reverse transcription polymerase chain reaction (RT-PCR), are the gold standard for respiratory pathogen detection, such as influenza viruses and SARS-CoV-2 [3,4]. These methods offer high sensitivity and specificity. However, NAATs are limited by high costs, prolonged processing, and requirements for specialized equipment and trained personnel, which constrain accessibility and flexibility—particularly during pandemics or in resource-limited settings. These constraints underscore the need for rational triage to narrow the differential diagnosis and prioritize targeted testing. Rapid antigen tests are commonly used in primary care settings without access to NAATs, or for home-based self-testing in suspected cases. While they offer rapid results, their sensitivity is generally lower and highly dependent on testing conditions [5,6]. In this context, a reliable, low-cost cross-check could not only help compensate for the sensitivity limitations of rapid tests but also serve as a potentially alternative tool for pathogen identification. Considering the limitations of current diagnostic methods, predictive models using easily obtainable clinical features (e.g., symptoms) have the potential to improve diagnostic timeliness and efficiency, enhance equitable access, and support targeted management while reducing unnecessary antibiotic use. Such a model is particularly valuable where laboratory capacity is limited, including low-resource settings, primary healthcare facilities, and outbreak situations.

In practice, respiratory symptoms or fever often serve as the initial trigger for pathogen testing in the diagnosis of URTIs, and symptoms represent some of the most readily obtainable clinical information associated with infection. Although URTIs are frequently characterized by nonspecific manifestations, pathogen-specific differences in pathogenesis and elicited host immune responses may shape distinct symptom profiles. While several studies have characterized the symptom profiles of specific pathogens [7–9], systematic comparative analyses across a broad spectrum of common respiratory viruses remain scarce.

Using self-reported symptom data and home-based multiplex testing results from individuals with upper respiratory symptoms in China, this study systematically characterized symptom signatures across common respiratory pathogens. We further developed a multilayer perceptron (MLP) model to evaluate whether symptom and demographic features could be leveraged to prioritize likely pathogens and support the narrowing of diagnostic testing.

## Results

### Study population

**Fig 1:**
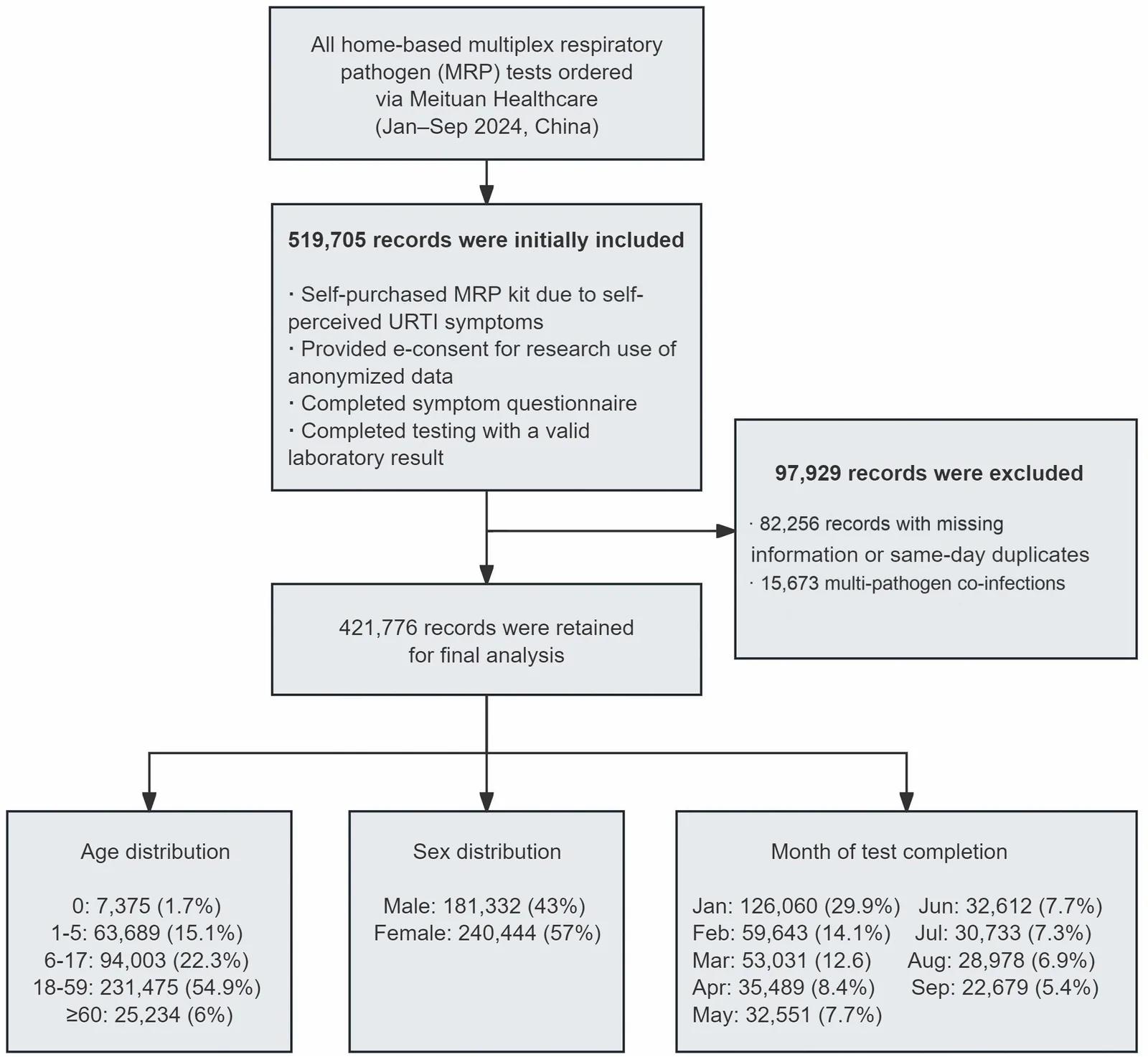
Data processing flowchart and baseline characteristics of the analytic data. The flow diagram summarizes inclusion and exclusion steps applied to the initial 519,705 records from January to September 2024, yielding a final analytic set of 421,776 single-pathogen infection samples with complete symptom data. The bottom panels show the characteristics of the final analytic cohort by age group, sex, and month of specimen collection; values are counts with percentages, reflecting the distribution among the 421,776 included samples.

An initial dataset of 519,705 records from January to September 2024 was extracted. After excluding records involving co-detection to minimize attribution bias that may confound pathogen-specific analyses, along with those containing incomplete information, duplicate same-day tests, or abnormal data, a total of 421,776 test results were retained for final analysis (Fig 1). These records were derived from individuals residing in Beijing (169,289, 40.1%), Chengdu (5,964, 1.4%), Guangzhou (41,524, 9.8%), Hangzhou (8,422, 2.0%), Nanjing (2,843, 0.7%), Shanghai (160,132, 38.0%), and Shenzhen (33,602, 8.0%). The median age of tested individuals was 30 years (interquartile range: 8–38 years), with females accounting for 57.0%. Age group distribution was as follows: infants (0 years), 1.7%; children (1–5 years), 15.1%; school-age adolescents (6–17 years), 22.3%; adults (18–59 years), 54.9%; and older adults (≥60 years), 6.0%. The majority of tested individuals were concentrated in the early months of the year, with January alone accounting for 29.9% of the total. The number of tested individuals then declined steadily over the months, reaching the lowest level in September, which accounted for only 5.4% of the total.

### Pathogen positivity patterns

In total, 37.9% (159,881/421,776) of participants were identified as single-pathogen positive, with SARS-CoV-2 (11.2%), Human adenovirus (HAdV) (5.5%), and Human rhinovirus (HRV) (5.0%) emerging as the three most frequently detected pathogens among the nine tested during the study period (S1 Table in Supporting Information). Clear seasonal dynamics were observed (S1 Fig in Supporting Information): Influenza B was initially the predominant virus in January; however, with the rapid rise in SARS-CoV-2 infections, the latter soon became the leading pathogen, reaching its first peak in March. After this initial peak, SARS-CoV-2 declined in March but resurged in late June, reaching a second peak in August. These trends are closely aligned with the temporal patterns of SARS-CoV-2 and influenza virus positivity rates among influenza-like illness (ILI) cases reported by national sentinel hospitals, as published in the Chinese Center for Disease Control and Prevention (China CDC) surveillance data [10]. Biweekly positivity rates of 9 tested respiratory pathogens stratified by sex and age group are available in S2 Fig and S3 Fig in Supporting Information.

Pathogen positivity rates varied notably by sex and age group (S1 Table in Supporting Information). Females exhibited significantly higher positivity rates for SARS-CoV-2 and respiratory syncytial virus (RSV), but lower rates for the other seven pathogens compared to males. Age-related differences were also observed. For example, the positivity of SARS-CoV-2 was markedly higher among older adults (≥60 years), while the positivity of HAdV peaked among young children (1–5 years) and school-aged individuals (6–17 years).

### Symptom signature of pathogen stratified by age and sex

We compared 13 clinical symptoms among patients infected with nine respiratory pathogens and further stratified by age and sex (Table 1 and S4 Fig in Supporting Information). Across pathogens, fever and cough were the most frequently reported symptoms, and overall symptom signatures showed broad overlap. However, differences in symptom distribution and severity were observed. For example, RSV, Human coronavirus (HCoV), Human parainfluenza virus (HPIV), and HRV were predominantly associated with respiratory symptoms, whereas influenza A, influenza B, and SARS-CoV-2 also showed more pronounced systemic symptoms, such as fever, headache, and myalgia. Moreover, HRV presented the lowest fever prevalence and rarely exhibited severe fever phenotypes (e.g., high-grade fever ≥39 °C or fever ≥3 days), whereas high-grade fever occurred more often in HAdV and influenza A.

**Table 1:** Age-specific symptom patterns in patients with single-pathogen infections.

Symptom and Pathogen	Symptom Distribution by Infecting Pathogen						*P* value
	All positive samples (N = 159881)	0 (n = 3078)	1-5 (n = 27096)	6-17 (n = 40536)	18-59 (n = 79845)	≥60 (n = 9326)	
**Influenza A**							
Fever	79.4	77.0	89.6	88.5	74.9	76.4	**<0.001**
Fever over 39°C	22.1	23.7	42.2	32.7	15.9	11.0	**<0.001**
Fever for 3 days	7.5	5.0	9.0	5.3	8.1	5.9	**<0.001**
Cough	64.8	66.2	58.4	58.2	67.6	67.8	**<0.001**
Sputum	23.1	17.3	17.1	18.9	26.5	18.8	**<0.001**
Sore throat	31.9	18.0	14.0	24.4	39.1	28.8	**<0.001**
Rhinorrhea	40.0	29.5	34.2	34.2	44.5	32.4	**<0.001**
Nasal congestion	29.9	21.6	23.0	27.6	34.0	19.6	**<0.001**
Dysphonia	14.3	10.1	6.9	8.1	18.4	11.1	**<0.001**
Headache	33.6	13.7	10.8	29.7	41.5	27.3	**<0.001**
Myalgia	33.6	13.7	8.0	16.4	45.3	31.6	**<0.001**
Diarrhoea	2.8	5.0	2.7	1.7	3.3	1.8	**<0.001**
Vomiting	5.9	7.9	11.7	9.7	3.6	4.1	**<0.001**
**Influenza B**							
Fever	74.4	77.2	91.8	87.6	67.9	57.6	**<0.001**
Fever over 39°C	14.3	15.5	30.6	24.4	8.7	5.2	**<0.001**
Fever for 3 days	11.2	7.8	10.7	8.3	12.6	5.8	**<0.001**
Cough	63.8	53.4	47.5	54.8	69.3	66.0	**<0.001**
Sputum	25.0	17.7	12.7	17.8	29.7	20.1	**<0.001**
Sore throat	34.9	15.5	11.8	22.8	43.0	28.2	**<0.001**
Rhinorrhea	45.7	37.1	32.7	33.6	52.4	36.4	**<0.001**
Nasal congestion	39.7	25.0	27.3	30.7	45.4	25.0	**<0.001**
Dysphonia	18.8	14.7	9.5	8.6	23.9	16.2	**<0.001**
Headache	35.0	18.5	10.6	28.2	41.6	22.2	**<0.001**
Myalgia	31.0	15.9	6.2	13.4	41.4	20.3	**<0.001**
Diarrhoea	3.1	1.7	2.1	1.9	3.8	1.2	**<0.001**
Vomiting	4.3	4.7	7.5	6.4	3.1	2.3	**<0.001**
**RSV**							
Fever	45.3	42.6	73.9	51.8	25.1	38.6	**<0.001**
Fever over 39°C	8.2	5.5	19.0	7.8	1.8	3.5	**<0.001**
Fever for 3 days	6.0	4.8	12.6	3.7	2.4	3.5	**<0.001**
Cough	74.4	84.8	85.1	70.2	66.7	73.3	**<0.001**
Sputum	35.9	40.8	41.2	30.2	33.7	30.2	**<0.001**
Sore throat	33.1	8.3	13.0	28.8	50.4	37.3	**<0.001**
Rhinorrhea	55.8	60.8	55.6	42.5	59.4	47.3	**<0.001**
Nasal congestion	47.4	45.6	38.8	40.5	56.2	39.8	**<0.001**
Dysphonia	21.6	13.8	16.2	10.8	28.4	22.5	**<0.001**
Headache	15.7	1.4	3.5	15.7	25.6	18.4	**<0.001**
Myalgia	9.4	0.7	1.6	5.5	16.2	14.1	**<0.001**
Diarrhoea	2.3	3.7	2.4	2.1	2.4	1.0	0.1
Vomiting	5.2	6.2	10.0	5.0	2.3	0.8	**<0.001**
**HCoV**							
Fever	55.8	60.0	77.1	66.1	26.0	45.4	**<0.001**
Fever over 39°C	9.4	9.4	17.9	9.4	1.9	2.5	**<0.001**
Fever for 3 days	1.7	3.5	2.6	1.4	0.9	3.4	**0.008**
Cough	45.3	48.2	40.8	47.9	45.4	57.1	**<0.001**
Sputum	14.7	14.1	12.5	14.9	16.4	15.1	0.14
Sore throat	28.5	17.6	12.1	30.6	42.8	28.6	**<0.001**
Rhinorrhea	39.1	28.2	27.6	32.6	56.5	46.2	**<0.001**
Nasal congestion	36.4	17.6	25.0	35.3	50.6	27.7	**<0.001**
Dysphonia	14.1	11.8	9.9	12.2	20.2	15.1	**<0.001**
Headache	16.0	3.5	5.1	18.6	24.3	18.5	**<0.001**
Myalgia	9.3	1.2	2.8	8.6	16.9	8.4	**<0.001**
Diarrhoea	2.0	3.5	2.0	1.2	2.7	0.8	0.08
Vomiting	3.5	3.5	5.1	4.3	1.4	1.7	**<0.001**
**HPIV**							
Fever	58.5	52.5	82.6	66.4	33.6	46.8	**<0.001**
Fever over 39°C	13.9	9.2	26.8	13.2	3.6	3.4	**<0.001**
Fever for 3 days	8.2	5.1	13.6	6.9	4.5	3.8	**<0.001**
Cough	62.0	67.1	55.9	65.2	63.8	79.5	**<0.001**
Sputum	26.1	25.4	20.0	24.6	31.7	34.8	**<0.001**
Sore throat	29.3	8.8	13.3	27.0	47.9	34.8	**<0.001**
Rhinorrhea	39.0	41.0	27.5	28.3	54.5	42.0	**<0.001**
Nasal congestion	34.3	26.8	20.3	29.3	52.1	27.3	**<0.001**
Dysphonia	19.6	11.9	14.7	13.0	27.8	23.5	**<0.001**
Headache	14.9	3.4	4.2	17.3	25.5	17.7	**<0.001**
Myalgia	10.2	0.7	2.5	7.8	19.8	12.6	**<0.001**
Diarrhoea	2.7	4.7	2.7	1.8	3.0	0.7	**0.01**
Vomiting	5.2	6.8	8.6	4.6	2.2	1.7	**<0.001**
**HRV**							
Fever	40.7	40.7	55.4	48.9	27.6	34.1	**<0.001**
Fever over 39°C	5.2	6.1	10.4	6.7	1.6	0.9	**<0.001**
Fever for 3 days	1.8	3.1	2.8	1.5	1.3	1.3	**<0.001**
Cough	54.8	54.1	59.3	55.1	51.6	62.2	**<0.001**
Sputum	20.4	17.1	18.6	18.6	22.6	20.4	**<0.001**
Sore throat	36.2	14.4	12.2	32.6	53.2	31.2	**<0.001**
Rhinorrhea	50.3	52.6	50.6	42.5	55.4	43.1	**<0.001**
Nasal congestion	44.8	40.4	39.2	41.4	51.1	30.3	**<0.001**
Dysphonia	14.0	6.1	7.7	9.1	21.0	15.9	**<0.001**
Headache	17.0	4.6	3.6	17.2	25.1	13.9	**<0.001**
Myalgia	10.0	1.5	1.6	6.5	17.2	10.8	**<0.001**
Diarrhoea	2.1	3.7	1.5	1.3	2.9	1.3	**<0.001**
Vomiting	3.3	3.4	5.1	4.5	1.6	1.6	**<0.001**
**SARS-CoV-2**							
Fever	60.3	77.5	84.9	78.6	53.3	63.3	**<0.001**
Fever over 39°C	7.3	20.4	23.3	14.5	4.4	4.1	**<0.001**
Fever for 3 days	2.7	3.7	3.1	1.5	2.7	3.7	**<0.001**
Cough	45.8	33.6	31.0	32.3	49.2	52.6	**<0.001**
Sputum	15.7	8.2	7.4	8.4	18.3	14.5	**<0.001**
Sore throat	41.9	14.9	11.6	30.1	49.9	31.2	**<0.001**
Rhinorrhea	39.3	28.1	32.1	31.0	42.7	34.6	**<0.001**
Nasal congestion	36.6	21.2	23.8	34.3	40.4	26.9	**<0.001**
Dysphonia	17.1	8.8	6.4	7.3	20.6	15.8	**<0.001**
Headache	28.9	8.1	6.3	24.3	33.7	22.4	**<0.001**
Myalgia	26.3	6.5	3.3	13.1	32.5	21.9	**<0.001**
Diarrhoea	2.4	2.5	2.1	1.2	2.8	1.3	**<0.001**
Vomiting	2.7	5.8	5.8	4.6	2.0	2.1	**<0.001**
**HAdV**							
Fever	74.8	73.4	83.9	83.1	56.7	41.4	**<0.001**
Fever over 39°C	26.5	20.4	36.9	31.9	10.7	4.2	**<0.001**
Fever for 3 days	12.8	10.6	15.4	14.6	8.4	5.1	**<0.001**
Cough	39.7	36.9	39.0	34.4	46.5	61.5	**<0.001**
Sputum	15.6	12.4	14.5	12.1	21.0	24.3	**<0.001**
Sore throat	29.0	14.2	14.9	25.8	47.5	32.3	**<0.001**
Rhinorrhea	18.7	18.2	20.6	16.0	20.7	19.8	**<0.001**
Nasal congestion	22.6	14.6	24.4	22.8	21.3	14.7	**<0.001**
Dysphonia	9.2	6.9	6.8	6.2	15.3	17.4	**<0.001**
Headache	21.1	9.1	10.3	25.0	26.9	16.7	**<0.001**
Myalgia	13.5	7.7	5.3	10.2	26.0	16.9	**<0.001**
Diarrhoea	3.8	3.6	4.0	3.7	3.9	1.3	0.07
Vomiting	8.3	7.7	10.6	10.4	3.6	1.3	**<0.001**
**M.pneumoniae**							
Fever	68.1	56.6	74.1	73.4	58.0	48.7	**<0.001**
Fever over 39°C	17.5	16.2	22.2	20.7	10.4	7.3	**<0.001**
Fever for 3 days	15.1	14.5	20.0	16.7	10.5	6.7	**<0.001**
Cough	71.5	71.5	74.7	70.4	72.0	68.5	**<0.001**
Sputum	27.5	28.5	26.8	26.7	29.9	21.2	**<0.001**
Sore throat	21.3	12.3	11.5	19.9	29.1	28.1	**<0.001**
Rhinorrhea	16.8	18.0	18.3	17.1	15.6	15.4	**0.02**
Nasal congestion	15.5	17.1	15.8	16.4	13.8	12.7	**<0.001**
Dysphonia	9.3	9.2	7.5	6.7	14.5	15.0	**<0.001**
Headache	19.4	11.4	6.5	18.8	27.9	20.5	**<0.001**
Myalgia	13.1	8.3	3.2	7.7	27.9	18.2	**<0.001**
Diarrhoea	2.4	3.1	2.4	2.5	2.3	2.1	0.88
Vomiting	5.5	7.9	7.3	6.9	2.3	1.2	**<0.001**

Age-stratified analysis revealed that, across the nine pathogens, most symptom distributions varied across age groups. Compared with adults aged 18–59 years, children and school-age adolescents (0–17 years) exhibited higher frequencies of fever-usually with greater severity and relatively lower frequencies of cough for most pathogens. Adults ≥60 years showed symptom distributions broadly similar to those of the 18–59 year group, but tended to report cough more often across many pathogens. Morever, symptoms such as sore throat, rhinorrhea, nasal congestion, and headache were generally less frequent in the 0–17 and ≥60-year groups than in the 18–59-year group. Sex-stratified analyses (S4 Fig in Supporting Information) revealed significant differences for certain symptoms, with the symptoms involved and the direction of effect varying by pathogen. Notably, high-grade fever (≥39°C) occurred more often in males than in females for nearly all pathogen groups, with the exception of *M. pneumoniae*.

Overall, symptom signatures largely overlapped across pathogens; observed differences were more categorical than pathogen-specific. Moreover, symptom expression was further influenced by age and sex.

### Symptom-based hierarchical clustering of respiratory pathogens and development of predictive models

Based on the similar features observed across some age groups, we further combined them in the main analysis into three categories: 0–5 years, 6–17 years, and ≥18 years, while the more detailed age stratifications were incorporated into the sensitivity analysis. In adults≥18 years, hierarchical clustering grouped the nine pathogens into three stable classes with similar symptom signature: Class 0-influenza A, influenza B, and SARS-CoV-2; Class 1-HAdV and *M. pneumoniae*; and Class 2-HCoV, HPIV, HRV, and RSV (Table 2). These clustering patterns were highly consistent across adult subgroups (18–45, 45–59, and ≥60 years). Clustering pattern in younger age groups showed some divergence from the adults group: in the 6–17 year group two pathogens were reassigned, with *M. pneumoniae* clustering with the influenza viruses and SARS-CoV-2 clustering with HAdV (Table 2). However, 0–5 years showed markedly different results (details provided in S5 Fig in Supporting Information), likely due to limited symptom self-reporting in this age group.

**Table 2:** Pathogen clustering based on symptom distribution and prediction performance of the MLP model.

Age Group	Class Category	Pathogen	Sensitivity (95%CI)	Specificity (95%CI)	AUC (95%CI)
0–5 years	Class 0	HAdV, HCoV, HPIV, Influenza A, Influenza B, SARS-CoV2	94.6% (92.9%–96.1%)	75.3% (73.0%–77.6%)	0.92 (0.91–0.93)
	Class 1	M. pneumoniae	53.1% (49.2%–56.5%)	99.4% (99.0%–99.8%)	0.86 (0.84–0.88)
	Class 2	HRV, RSV	75.1% (71.5%–78.5%)	86.9% (85.1%–88.8%)	0.91 (0.89–0.92)
6–17 years	Class 0	Influenza A, Influenza B, M. pneumoniae	60.4% (58.1%–62.8%)	81.2% (80.0%–82.5%)	0.83 (0.82–0.84)
	Class 1	HAdV, SARS-CoV2	66.5% (64.2%–68.6%)	75.3% (74.0%–76.9%)	0.80 (0.78–0.81)
	Class 2	HCoV, HPIV, HRV, RSV	58.4% (56.1%–60.8%)	86.1% (85.0%–87.2%)	0.81 (0.80–0.83)
≥18 years	Class 0	Influenza A, Influenza B, SARS-CoV2	97.7% (97.1%–98.3%)	74.8% (73.8%–76.0%)	0.91 (0.91–0.92)
	Class 1	HAdV, M. pneumoniae	57.7% (55.8%–59.5%)	94.4% (93.8%–95.0%)	0.85 (0.85–0.86)
	Class 2	HCoV, HPIV, HRV, RSV	62.3% (60.5%–64.1%)	89.7% (88.9%–90.6%)	0.87 (0.86–0.88)

**Fig 2:**
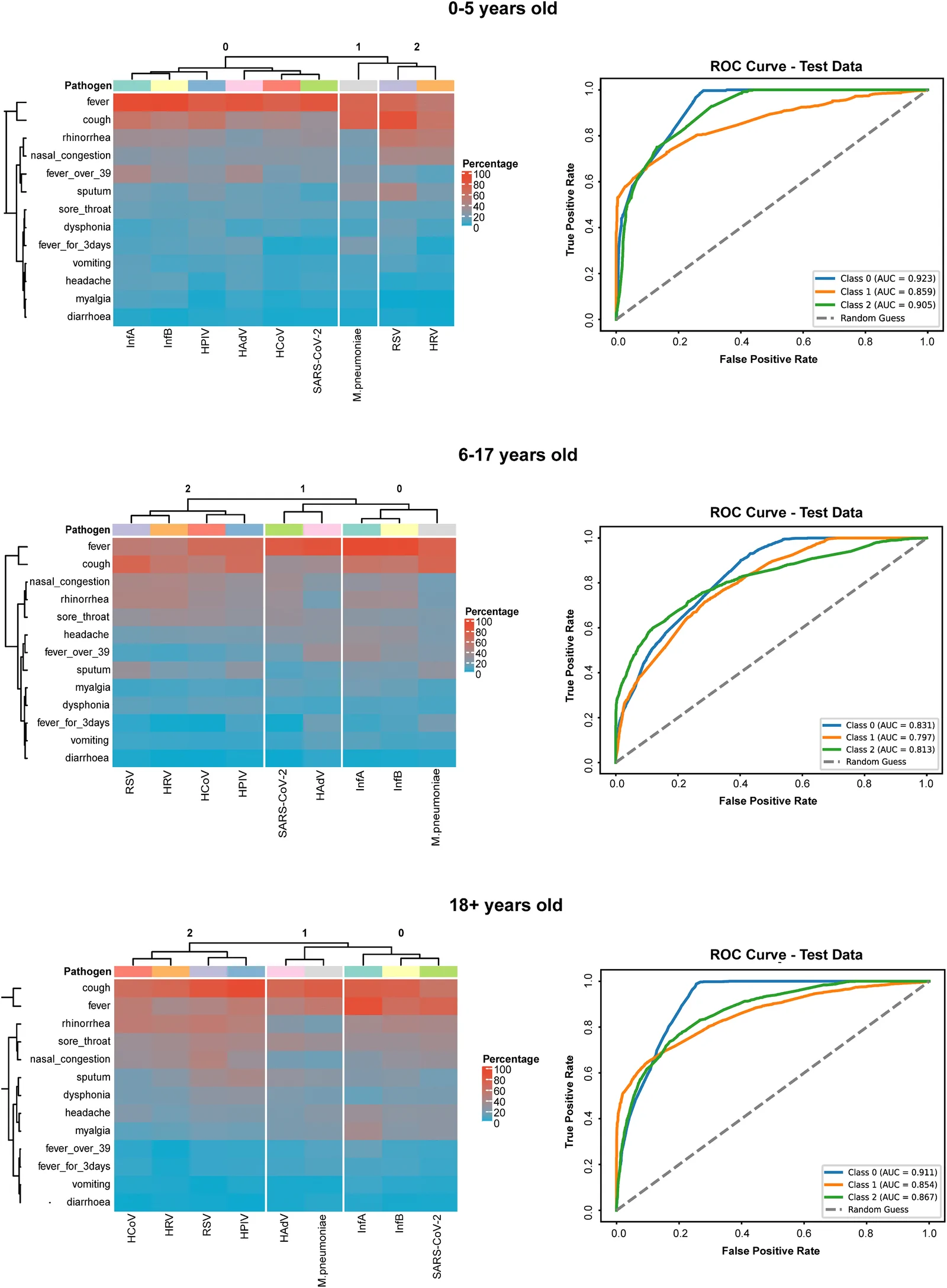
Symptom-based pathogen class definition and MLP classification performance stratified by three age groups. The left panels show hierarchical clustering of nine pathogens into three classes (labeled 0, 1, and 2 at the top) based on their symptom profile distributions within each of three age groups. Each column represents a specific pathogen and each row represents a symptom, with the color gradient from blue to red indicating increasing prevalence of the symptom in infections by specific pathogen. The right panels depict the performance of a MLP model trained to classify individual infections into these three predefined classes: one-vs-rest ROC curves are shown for each age group, with associated AUC values annotated, reflecting the model’s ability to differentiate symptom-defined infections across pathogen classes.

We then trained a MLP model to predict the broad pathogen classes from symptoms (Table 2). Although the clustering was entirely data-driven, certain classes showed relatively consistent symptom patterns (Fig 2), which were highly aligned with the features identified earlier section. For example, in both the 6–17 and ≥18 years age groups, HCoV, HPIV, HRV, and RSV were clustered together, and these infections tended to produce typical upper respiratory symptoms such as rhinorrhea, cough, and sore throat, with a relatively lower prevalence of systemic symptoms compared with the other classes. In contrast, infections with influenza A, influenza B, SARS-CoV-2, and *M. pneumoniae*-although the clustering of SARS-CoV-2 and *M. pneumoniae* varied slightly across age groups—were consistently characterized by more systemic manifestations and higher rates of fever.

The MLP model demonstrated overall good ability to distinguish broad pathogen classes, broad pathogen classes, with area under the curve (AUC) values of 0.85-0.91 in adults≥18 years, 0.86-0.92 in infants and children aged 0–5 years, and slightly lower values of 0.80-0.83 in those aged 6–17 years (Table 2). Calibration plots suggested reasonable agreement between predicted and observed probabilities (S6 Fig in Supporting Information). Notably, the model exhibited a conservative classification tendency across most class categories, with specificity all above 75%, but relatively lower sensitivity in several classes. For example, in the ≥ 18 years group, Class 1 (HAdV and *M. pneumoniae*) had a sensitivity as low as 57.7%. In the 0–5 years group, Class 1 (*M. pneumoniae*) had a sensitivity of only 53.1%. The high AUCs and specificities but low sensitivities indicate a tendency to under-detection. Incorporating epidemiological information may help improve case identification.

### Refining predictions of each pathogen by integrating epidemiological data

**Fig 3:**
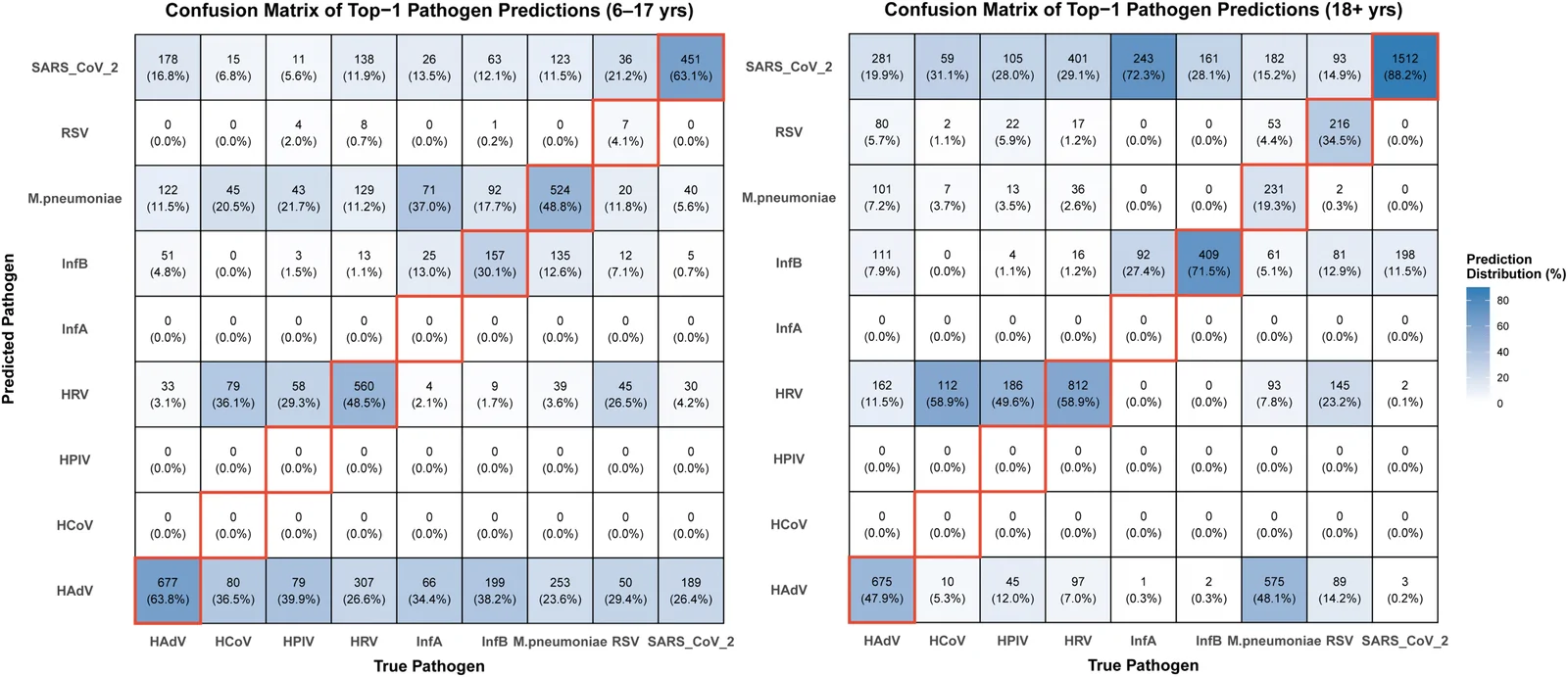
Confusion matrix of MLP top-1 pathogen predictions after integration of epidemiological data. This figure shows the confusion matrix of top-1 pathogen predictions in the 6–17 and 18+ years-old age groups, after post-hoc reweighting of MLP model outputs with epidemiological data. Each cell shows both the number of samples and the percentage of all cases with that true pathogen that were predicted as the corresponding pathogen. Cells are shaded from white to blue according to the prediction distribution percentage, with darker indicating higher proportions. Red outlines highlight the diagonal cells representing correct predictions.

Fig 3 illustrates the comparison between the post-hoc reweighted MLP predictions–adjusted using pathogen epidemiological data–with the observed pathogen distributions across age groups. When only the top-ranked predicted pathogen was used as the final classification, the overall accuracy was only 0.45 [95% CI, 0.43-0.46] (κ = 0.32 [95%CI, 0.31-0.32]) in 6–17 years group and 0.49 [95% CI, 0.48-0.51] (κ = 0.39 [95%CI, 0.37-0.39]) in individuals ≥18 years. Regarding prediction of individual pathogens, the model achieved moderate accuracy for some pathogens (S2 Table in Supporting Information). For example, in the 6–17 year old age group, the model achieved a sensitivity of 0.64 (95% CI: 0.61-0.67) and a specificity of 0.71 (95% CI: 0.70-0.73) for detecting HAdV. Among adults aged≥18 years, the model demonstrated high sensitivity for identifying SARS-CoV-2, reaching 0.88 (95% CI: 0.87–0.90). However, the model demonstrated little to no capacity to distinguish certain pathogens—including HCoV, HPIV, influenza A, and RSV—with sensitivities approaching zero. Expanding the predicted target to include the top two most probable pathogens improved overall performance. The classification accuracy increased to 0.67 (95%CI, 0.66-0.69) in aged 6–17 and 0.71(95%CI, 0.70-0.72) in those ≥18 years. Further extending the candidate range to the top three predictions resulted in an accuracy of 0.83 (95%CI, 0.82,0.84) for 6–17-year age group and 0.83 (95%CI, 0.82,0.83) for ≥18-year-olds. Overall, integration of epidemiological data enhanced predictions for prevalent pathogens but not for less common ones; however, broadening the prediction to include the top two or three likely pathogens markedly improved overall accuracy.

## Discussion

In this study, we utilized a large-scale home-based self-testing dataset and machine learning models to systematically characterize the symptom signature associated with nine common respiratory pathogens across different age and sex groups. Despite the broad overlap in overall symptom profiles, distinct symptom-based categories were identified. For example, RSV, HCoV, HPIV, and HRV were predominantly associated with respiratory symptoms, whereas influenza A/B and SARS-CoV-2 exhibited more pronounced systemic manifestations. An multilayer perceptron model trained on hierarchical-cluster labels achieved high AUCs (0.80–0.92, depending on age group) with specificities >75% but lower sensitivities, indicating a conservative, under-detecting tendency. Integrating cluster-level predictions with contemporaneous pathogen-specific epidemiological data improved the prioritization of likely pathogens for prevalent infections, while performance remained limited for less common pathogens. Nonetheless, the model helped the narrowing of diagnostic testing, with accuracy of 0.83 (95% CI, 0.82–0.84) when expanding predictions to the top three likely pathogens.

Symptom-related patient-reported outcomes (PROs) are increasingly incorporated into clinical trials evaluating antiviral therapies, in alignment with guidance from regulatory agencies such as the U.S. Food and Drug Administration (FDA) [11]. In addition to conventional endpoints such as the frequency of symptom exacerbation or patient-reported time to symptom alleviation [12–14], validated symptom-based PRO instruments have been widely adopted, including FLU-PRO and Flu-iiQ for influenza-like illness [15,16] and RiiQ for RSV [17]. However, the application of these standardized PRO tools remains limited for other acute URTIs caused by a broader range of pathogens. By systematically characterizing symptom profiles across nine common respiratory pathogens, our study provides clinicians and researchers with a clearer understanding of pathogen-associated symptom patterns. This uniform evaluation also offers high-quality evidence to support the broader application of symptom-related PROs. Specifically, for pathogens whose primary symptoms closely overlap with influenza and SARS-CoV-2, existing symptom scales may be considered for direct application, as the established domains generally capture their principal symptomatology. However, for pathogens exhibiting distinct symptom characteristics, targeted adjustments—such as adding or removing specific items—may enhance the efficiency, sensitivity, and utility of these PRO tools in both clinical research and practice.

Our study identified heterogeneity in the frequency and severity of symptoms across different age and sex groups. These findings underscore the importance of ensuring demographic balance when using symptom-related PROs in clinical research. Such variability may otherwise confound the interpretation of treatment effects or limit generalizability. Moreover, the biological mechanism of these disparities remains unclear but may involve differences in immune response, hormonal regulation, or behavioral factors, highlighting the need for further mechanistic investigation.

In exploring the use of symptoms and other readily obtainable clinical features to support URTIs diagnostic testing decisions, most existing studies have focused on predicting a single target pathogen rather than distinguishing among multiple common respiratory pathogens [18]. This single-pathogen prediction framework has clear limitations for clinical application: in real-world initial assessments, clinicians must navigate a complex landscape of multiple possible pathogens. Within the published literature, we identified only four original studies that attempted pathogen distinguishment using symptoms and other readily accessible clinical information. Three of these compared only two pathogens [19–21], while the study by Nadda et al., used symptom descriptions and patient information from outpatient records to distinguish the common cold, influenza, and dengue [22]. These studies covered a narrow pathogen spectrum and did not reflect the multi-pathogen diagnostic scenarios common in clinical practice, nor did they assess symptom patterns across different ages or settings. Moreover, existing models generally rely on structured electronic medical records or clinic-based data, with little characterization of symptom profiles in community-based or home-testing contexts. In this context, our study provides insights that may assist in addressing this gap in diagnostic testing prioritization and stewardship. By combining a multilayer MLP model with real-time prevalence data, our findings demonstrate that structured symptom information can support the prioritization of diagnostic testing targets during initial assessment, potentially reducing unnecessary testing and improving resource allocation. Nevertheless, further validation of this machine learning–based pathogen prediction approach is required in real-world healthcare settings and through health-economic evaluations.

The practical utility of our approach relies on timely access to high-quality epidemiological surveillance data for upper respiratory infections and early alerts to emerging or rising infections. China has established national surveillance networks for monitoring pathogens such as influenza, RSV, SARS-CoV-2, and clinical syndromes including influenza-like illness (ILI), pneumonia, and severe acute respiratory infections (SARI) [23,24]. However, sentinel surveillance data derived from healthcare institutions suffer from limited pathogen coverage and lack representativeness of the general population. In addition, publicly available data often lack the detail, timeliness, and accessibility required for real-time clinical use. Given these limitations, we advocate for improved data-sharing practices and greater transparency in reporting. Additionally, the close alignment between home-based self-testing results in our study and official CDC data highlights the potential for such user-generated data to serve as a valuable complement to existing surveillance systems.

Several limitations should be considered. First, although the temporal trends in pathogen positivity observed in our study were largely consistent with those reported by China CDC, the self-test positivity rates presented here are inevitably subject to selection bias related to socioeconomic status, health awareness, and accessibility to testing. Second, although the sample size is large, the data—collected from January to September 2024 across seven cities—are not nationally representative. The exclusive focus on home-based testing further limits generalizability to more severe cases requiring clinical care. Third, the study relied on patient-reported symptoms, which are inherently subjective and may compromise data quality. This limitation is particularly pronounced in pediatric populations, where symptom reporting is often imprecise due to communication barriers. Fourth, the available patient information was limited, lacking important variables including comorbidities, smoking history, and medication use. The limited availability of detailed patient-level clinical data, combined with considerable overlap of clinical symptoms across multiple respiratory pathogens, resulted in relatively modest pathogen distingushing performance using machine learning models. Fifth, although multiplex PCR can detect multiple pathogens, we restricted the analysis to single-pathogen infections and excluded co-infected cases during model training. While this approach allowed for clearer characterization of pathogen-specific symptom patterns, it may limit generalizability, as symptom patterns in co-infections can be more complex and are not captured by our model. Moreover, integrating population-level epidemiological data did not substantially improve overall prediction accuracy and, due to weighting toward more prevalent pathogens, even reduced accuracy for less common ones. Last, this study focused prioritizing diagnostic targets among multiple pathogens based on symptom profiles, rather than addressing the earlier clinical question of whether testing is needed. As a result, its applicability is largely confined to clinical situations in which testing has already been deemed necessary and the key question is which pathogens should be prioritized for diagnostic evaluation.

Our findings have several practical implications for both clinical and public health settings. First, this approach can support the prioritization of diagnostic testing. By integrating patients’ symptom profiles with real-time epidemiological data, the model could generate a ranked list of likely pathogens, thereby narrowing the diagnostic scope and guiding targeted testing before multiplex assays (e.g., PCR). This may improve testing efficiency, reduce unnecessary investigations, and optimize resource allocation. Second, in low-resource settings or outbreak situations where laboratory capacity is limited or results are delayed, the model can provide early risk stratification based on symptom patterns, supporting rapid triage and initial empirical decision-making. Such applications may enhance emergency response efficiency, alleviate pressure on healthcare systems, and improve equity in access to timely diagnostic support. The model could be deployed as a web-based tool, enabling its use within routine clinical and public health workflows. Effective implementation would require regular updating with local epidemiological data and further validation in real-world healthcare settings. Importantly, this approach is intended to complement, rather than replace, multiplex laboratory testing, particularly when testing resources are constrained or results are pending.

Overall, the stratified symptom profiles from large-scale, home-based multiplex respiratory pathogen (MRP) testing clarify the similarities and differences in symptom presentation across pathogens, enhancing our understanding of age- and sex-specific clinical patterns. These findings also provide a foundation for the symptom-based, machine learning–assisted tools to support the early assessment of upper respiratory tract infections. With further validation in real-world healthcare settings, this approache could contribute to more targeted diagnostic triage, more efficient use of testing resources, and improved decision-making during the early assessment of upper respiratory tract infections, especially in outbreak settings or resource-limited environments where timely laboratory testing may be unavailable.

## Materials and methods

### Ethics statement

The study protocol was reviewed and approved by the Ethics Committee of China-Japan Friendship Hospital (Approval No. 2024-KY-436). The data used in this study were obtained through a research collaboration with Meituan Healthcare (Beijing, China), which provided anonymized respiratory specimen and pathogen testing data under a national research project for research only. Meituan had no role in the study design, data interpretation, or manuscript preparation. All participants provided electronic informed consent for the use of anonymized testing data for scientific research purposes. For the present study, only fully anonymized secondary data were analyzed, and no identifiable personal information was accessed by the researchers.

### Study design and data source

A cross-sectional study was conducted based on data from Meituan Healthcare (Beijing, China), a nationwide pharmaceutical e-commerce platform. The study population comprised individuals who self-purchased multiplex respiratory pathogen (MRP) testing kits due to self-perceived upper respiratory tract infection symptoms and opted for self-testing via the platform. Before ordering the testing kits, participants were required to provide informed consent for the use of anonymized testing data in scientific research and complete a brief questionnaire collecting information on sex, age, and symptoms. Reported symptoms included fever, fever≥ 39 °C, fever lasting≥ three days, cough, sputum production, sore throat, rhinorrhea, nasal congestion, dysphonia, headache, myalgia, diarrhoea, and vomiting. For young children unable to complete the ordering process independently, parents could purchase the testing service on their behalf, provide the necessary personal information, and assist with sample collection. The samples were transported to laboratories accredited by the China National Accreditation Service for Conformity Assessment (CNAS) by trained delivery personnel. Polymerase chain reaction (PCR) testing was conducted to simultaneously detect nine pathogens: influenza A, influenza B, RSV, HRV, HPIV, HAdV, HCoV, *M. pneumoniae*, and SARS-CoV-2.

### Data management and statistical analysis

The study included participants who conducted self-testing, provided informed consent, and received test results between January 1 and September 30, 2024. Their test results, along with self-reported sex, age, and symptoms from the questionnaire, were extracted from the Meituan Healthcare platform. Due to the accessibility of test kit purchases, participants were primarily from seven major cities in China: Beijing, Chengdu, Guangzhou, Hangzhou, Nanjing, Shanghai, and Shenzhen. Individuals with missing symptom data or test results were excluded. Additionally, to minimize the influence of co-infections on symptom pattern analysis, individuals with multi-pathogen infections were excluded.

Five age groups were defined for analysis: infants (0 years), children (1–5 years), school-age individuals (6–17 years), adults (18–59 years), and older adults (≥60 years). We examined changes in the detection rates of upper respiratory tract pathogens across different age and sex groups over the study period, defined as the proportion of individuals with single-pathogen infections among all tested individuals. We restricted the symptom analysis to single-pathogen infections and compared symptom profiles across pathogens. Individuals with co-detection were excluded to avoid attribution bias, which can confound pathogen-specific comparisons and degrade model performance. Categorical variables were summarized using counts and percentages. The chi-square test was employed to assess differences in qualitative variables, and differences between proportions were evaluated using the two-sample proportion test. *P* values were adjusted for multiple comparisons using the Bonferroni method, and a two-sided *P* value of <0.05 was considered statistically significant.

### Symptom-based clustering and MLP modeling

To simplify the classification task and investigate the feasibility of symptom-based pathogen prediction, we first applied hierarchical clustering to group the nine pathogens into three broader classes based on their age-stratified symptom distribution patterns. This class-merging strategy led to improved classification performance in preliminary experiments, outperforming direct nine-class classification models in accuracy and stability. For the three-class classification task, we developed an MLP model comprising three fully connected layers. Each layer was followed by a ReLU activation function to introduce non-linearity, and a dropout layer with a dropout probability of 0.5 to prevent overfitting. The model was optimized using the Adam algorithm with an initial learning rate of 1e-4, β₁ = 0.9, β_2_ = 0.999, and a weight decay of 0.001.

To address class imbalance in the training data, we employed a k-means–based downsampling strategy, where samples from the majority classes were clustered and downsampled to match the size of the minority class, ensuring a balanced dataset across all categories. The final dataset of single-pathogen infection cases was randomly split into training (60%), validation (20%), and test (20%) sets. Model training was conducted for a maximum of 100 epochs, and the model with the lowest validation loss was selected as the final model. Performance on the test set was evaluated using sensitivity, specificity, and area under the receiver operating characteristic curve (AUC), with 95% confidence intervals estimated using bootstrap resampling. Model calibration was assessed by calibration plots comparing predicted probabilities with observed event proportions for each class across age groups.

### Integrating epidemiological data to enhance MLP predictions

To assess whether incorporating epidemiological data can improve pathogen identification, we performed a post-hoc refinement of MLP predictions. Based on the predicted probabilities of the three broader pathogen classes generated by the MLP, we first assigned equal probabilities to each pathogen within a given class. These initial probabilities were then adjusted using the two-week detection rates of each pathogen at the population level. The weighted probabilities were normalized to sum to one for each case, and the pathogen with the highest adjusted probability was taken as the predicted result. The pathogen with the highest final probability was taken as the predicted result. Prediction performance was assessed using multiple complementary metrics. Overall agreement between predicted and true pathogen classes was quantified by classification accuracy and Cohen’s Kappa coefficient, while per-pathogen diagnostic performance was characterized by sensitivity, specificity, precision, and F1-score, each with 95% confidence intervals. Statistical analysis was performed with R software, version 4.3.2.

## Supporting information

S1 Fig Biweekly positive rates of nine tested respiratory pathogens. (DOCX)

S2 Fig Biweekly positive rates of nine respiratory pathogens stratified by age group. (DOCX)

S3 Fig Biweekly positive rates of nine respiratory pathogens stratified by sex. (DOCX)

S4 Fig Age- and sex-stratified symptom profiles among single-pathogen infections. (DOCX)

S5 Fig Symptom-based pathogen class definition and MLP classification performance stratified by six age groups. (DOCX)

S6 Fig Calibration plots of the three-class classification model across age groups. (DOCX)

S1 Table Positivity rates of different pathogens stratified by sex and age group. (DOCX)

S2 Table Performance of pathogen-level predictions stratified by age group after integration of epidemiological data. (DOCX)
