# Supplementary material for: Distinguishing common respiratory pathogens using machine learning of symptom profiles to prioritize diagnostic testing
Source: PLOS Digit Health. 2026 Aug 21;5(8):e0001656. doi: 10.1371/journal.pdig.0001656 (PMC13496429; doi:10.1371/journal.pdig.0001656)
Supplement: Supplementary file 1 [file pdig.0001656.s001.docx]

**S1 Fig. Biweekly positive rates of nine tested respiratory pathogens**

This figure shows the temporal dynamics of test positive rates for nine respiratory pathogens over the study period in two-week intervals. Positive rate is defined as the number of positive detections for a given pathogen divided by the total number of tests performed in the same interval. Lines in different colors represent different pathogens.
