# Supplementary material for: Distinguishing common respiratory pathogens using machine learning of symptom profiles to prioritize diagnostic testing
Source: PLOS Digit Health. 2026 Aug 21;5(8):e0001656. doi: 10.1371/journal.pdig.0001656 (PMC13496429; doi:10.1371/journal.pdig.0001656)
Supplement: Supplementary file 2 [file pdig.0001656.s002.docx]

**S2 Fig. Biweekly positive rates of nine respiratory pathogens stratified by age group**

This figure shows the temporal dynamics of test positive rates for nine respiratory pathogens over the study period in two-week intervals. Positive rate is defined as the number of positive detections for a given pathogen divided by the total number of tests performed in the same interval. Colored lines represent age groups: infants (0 years), young children (1–5 years), school-age individuals (6–17 years), adults (18–59 years), and older adults (>=60 years).

InfA, Influenza A; InfB, Influenza B; RSV, respiratory syncytial virus; HCoV, human coronavirus; HPIV, human parainfluenza virus; HRV, human rhinovirus; SARS-CoV-2, severe acute respiratory syndrome coronavirus 2; HAdV, human adenovirus; M. pneumoniae, Mycoplasma pneumoniae.
