# Supplementary material for: Distinguishing common respiratory pathogens using machine learning of symptom profiles to prioritize diagnostic testing
Source: PLOS Digit Health. 2026 Aug 21;5(8):e0001656. doi: 10.1371/journal.pdig.0001656 (PMC13496429; doi:10.1371/journal.pdig.0001656)
Supplement: Supplementary file 3 [file pdig.0001656.s003.docx]

**S3 Fig. Biweekly positive rates of nine respiratory pathogens stratified by sex**

This figure shows the temporal trends in test positive rates for nine respiratory pathogens over the study period in two-week intervals, stratified by sex. Positive rate is defined as the number of positive detections for a given pathogen divided by the total number of tests performed in the same interval. Lines in different colors represent sex groups: Blue lines represent males and orange lines represent females.

InfA, Influenza A; InfB, Influenza B; RSV, respiratory syncytial virus; HCoV, human coronavirus; HPIV, human parainfluenza virus; HRV, human rhinovirus; SARS-CoV-2, severe acute respiratory syndrome coronavirus 2; HAdV, human adenovirus; M. pneumoniae, Mycoplasma pneumoniae.
