# Supplementary material for: Distinguishing common respiratory pathogens using machine learning of symptom profiles to prioritize diagnostic testing
Source: PLOS Digit Health. 2026 Aug 21;5(8):e0001656. doi: 10.1371/journal.pdig.0001656 (PMC13496429; doi:10.1371/journal.pdig.0001656)
Supplement: Supplementary file 4 [file pdig.0001656.s004.docx]

**S4 Fig. Age- and sex-stratified symptom profiles among single-pathogen infections**

This figure displays the distributions of 13 symptoms (fever, fever over 39°C, fever lasting three days, cough, sputum production, sore throat, rhinorrhea, nasal congestion, dysphonia, headache, myalgia, diarrhea, and vomiting) for infections with each of nine pathogens, stratified by age and sex groups. Bar plots and accompanying values represent the proportion of individuals infected with a given pathogen who exhibit specific symptom (i.e., number of cases with specific pathogen and symptom divided by total infections with that pathogen). Colors of the bars indicate sex: blue for males and orange for females.

InfA, Influenza A; InfB, Influenza B; RSV, respiratory syncytial virus; HCoV, human coronavirus; HPIV, human parainfluenza virus; HRV, human rhinovirus; SARS-CoV-2, severe acute respiratory syndrome coronavirus 2; HAdV, human adenovirus; M. pneumoniae, Mycoplasma pneumoniae.
