# Supplementary material for: Distinguishing common respiratory pathogens using machine learning of symptom profiles to prioritize diagnostic testing
Source: PLOS Digit Health. 2026 Aug 21;5(8):e0001656. doi: 10.1371/journal.pdig.0001656 (PMC13496429; doi:10.1371/journal.pdig.0001656)
Supplement: Supplementary file 5 [file pdig.0001656.s005.docx]

**S5 Fig. Symptom-based pathogen class definition and MLP classification performance stratified by six age groups**

The left panels show hierarchical clustering of nine pathogens into three classes (labelled as 0, 1, and 2 at the top) based on their symptom profile distributions within each of six age groups. Each column represents a specific pathogen and each row represents a symptom, with the color gradient from blue to red indicating increasing prevalence of the symptom in infections by specific pathogen. The right panels depict the performance of a multilayer perceptron (MLP) trained to classify individual infections into these three predefined classes: one-vs-rest ROC curves are shown for each age group, with associated AUC values annotated, reflecting the model’s discriminative ability to assign symptom-defined infections to the correct class.

InfA, Influenza A; InfB, Influenza B; RSV, respiratory syncytial virus; HCoV, human coronavirus; HPIV, human parainfluenza virus; HRV, human rhinovirus; SARS-CoV-2, severe acute respiratory syndrome coronavirus 2; HAdV, human adenovirus; M. pneumoniae, Mycoplasma pneumoniae.
