# Supplementary material for: Distinguishing common respiratory pathogens using machine learning of symptom profiles to prioritize diagnostic testing
Source: PLOS Digit Health. 2026 Aug 21;5(8):e0001656. doi: 10.1371/journal.pdig.0001656 (PMC13496429; doi:10.1371/journal.pdig.0001656)
Supplement: Supplementary file 6 [file pdig.0001656.s006.docx]

**S6 Fig. Calibration plots of the three-class classification model across age groups**

This figure presents calibration plots comparing predicted probabilities with observed proportions for the three-class classification model across different age groups (0–5 years, 6–17 years, and ≥18 years). The dashed diagonal line represents perfect calibration, where predicted probabilities equal observed outcomes. Colored lines correspond to the three classes (Class 0, Class 1, and Class 2). The mapping between classes and pathogens across age groups is provided in Figure 2 and Table 2.
