# Supplementary material for: Distinguishing common respiratory pathogens using machine learning of symptom profiles to prioritize diagnostic testing
Source: PLOS Digit Health. 2026 Aug 21;5(8):e0001656. doi: 10.1371/journal.pdig.0001656 (PMC13496429; doi:10.1371/journal.pdig.0001656)
Supplement: Supplementary file 7 [file pdig.0001656.s007.docx]

**S1 Table. Positivity rates of different pathogens stratified by sex and age group**

| **Pathogen** | **Total**  **(n=421776)** | **Gender** | | | **Age Group** | | | | | |
| --- | --- | --- | --- | --- | --- | --- | --- | --- | --- | --- |
|  |  | **Male**  **(n=181332)** | **Female**  **(n=240444)** | **P value** | **0**  **(n=7375)** | **1-5**  **(n=63689)** | **6-17**  **(n=94003)** | **18-59**  **(n=231475)** | **≥60**  **(n=25234)** | **P value** |
| Influenza A | 10798 (2.6%) | 4811 (2.7%) | 5987 (2.5%) | **＜0.001** | 139 (1.9%) | 1532 (2.4%) | 1797 (1.9%) | 6376 (2.8%) | 954 (3.8%) | **＜0.001** |
| Influenza B | 20595 (4.9%) | 9095 (5%) | 11500 (4.8%) | **＜0.001** | 232 (3.1%) | 1797 (2.8%) | 4816 (5.1%) | 13233 (5.7%) | 517 (2%) | **＜0.001** |
| RSV | 8039 (1.9%) | 3284 (1.8%) | 4755 (2%) | **＜0.001** | 434 (5.9%) | 2588 (4.1%) | 805 (0.9%) | 3702 (1.6%) | 510 (2%) | **＜0.001** |
| HCoV | 3594 (0.9%) | 1506 (0.8%) | 2088 (0.9%) | 0.19 | 85 (1.2%) | 1095 (1.7%) | 1144 (1.2%) | 1151 (0.5%) | 119 (0.5%) | **＜0.001** |
| HPIV | 6195 (1.5%) | 2803 (1.5%) | 3392 (1.4%) | **＜0.001** | 295 (4%) | 2266 (3.6%) | 1033 (1.1%) | 2308 (1%) | 293 (1.2%) | **＜0.001** |
| HRV | 21293 (5%) | 9587 (5.3%) | 11706 (4.9%) | **＜0.001** | 327 (4.4%) | 5269 (8.3%) | 5874 (6.2%) | 9268 (4%) | 555 (2.2%) | **＜0.001** |
| SARS-CoV-2 | 47393 (11.2%) | 17955 (9.9%) | 29438 (12.2%) | **＜0.001** | 1064 (14.4%) | 3068 (4.8%) | 6165 (6.6%) | 31732 (13.7%) | 5364 (21.3%) | **＜0.001** |
| HAdV | 23095 (5.5%) | 11135 (6.1%) | 11960 (5%) | **＜0.001** | 274 (3.7%) | 6397 (10%) | 9334 (9.9%) | 6641 (2.9%) | 449 (1.8%) | **＜0.001** |
| M.pneumoniae | 18879 (4.5%) | 8984 (5%) | 9895 (4.1%) | **＜0.001** | 228 (3.1%) | 3084 (4.8%) | 9568 (10.2%) | 5434 (2.3%) | 565 (2.2%) | **＜0.001** |

RSV, respiratory syncytial virus; HCoV, human coronavirus; HPIV, human parainfluenza virus; HRV, human rhinovirus; SARS-CoV-2, severe acute respiratory syndrome coronavirus 2; HAdV, human adenovirus; M. pneumoniae, Mycoplasma pneumoniae.
