# Supplementary material for: Distinguishing common respiratory pathogens using machine learning of symptom profiles to prioritize diagnostic testing
Source: PLOS Digit Health. 2026 Aug 21;5(8):e0001656. doi: 10.1371/journal.pdig.0001656 (PMC13496429; doi:10.1371/journal.pdig.0001656)
Supplement: Supplementary file 8 [file pdig.0001656.s008.docx]

**S2 Table. Performance of pathogen-level predictions** **stratified by age group after integration of epidemiological data**

| **Age Group** | **Pathogen** | **Sensitivity (95%CI)** | **Specificity (95%CI)** | **Precision (95%CI)** | **F1 (95%CI)** |
| --- | --- | --- | --- | --- | --- |
| 6-17 years | HAdV | 0.64 (0.61–0.67) | 0.71 (0.70–0.73) | 0.36 (0.34–0.38) | 0.46 (0.43–0.48) |
|  | HCoV | 0.00 (0.00–0.02) | 1.00 (1.00–1.00) | - | - |
|  | HPIV | 0.00 (0.00–0.02) | 1.00 (1.00–1.00) | - | - |
|  | HRV | 0.48 (0.46–0.51) | 0.93 (0.92–0.94) | 0.65 (0.62–0.68) | 0.56 (0.53–0.58) |
|  | Influenza A | 0.00 (0.00–0.02) | 1.00 (1.00–1.00) | - | - |
|  | Influenza B | 0.30 (0.26–0.34) | 0.95 (0.94–0.95) | 0.39 (0.34–0.44) | 0.34 (0.30–0.38) |
|  | M.pneumoniae | 0.49 (0.46–0.52) | 0.87 (0.86–0.88) | 0.48 (0.45–0.51) | 0.49 (0.46–0.51) |
|  | RSV | 0.04 (0.02–0.08) | 1.00 (1.00–1.00) | 0.35 (0.18–0.57) | 0.07 (0.02–0.12) |
|  | SARS_CoV_2 | 0.63 (0.59–0.67) | 0.87 (0.86–0.88) | 0.43 (0.40–0.46) | 0.51 (0.48–0.54) |
| ≥18 years | HAdV | 0.48 (0.45–0.50) | 0.87 (0.86–0.88) | 0.45 (0.43–0.48) | 0.46 (0.44–0.49) |
|  | HCoV | 0.00 (0.00–0.02) | 1.00 (1.00–1.00) | - | - |
|  | HPIV | 0.00 (0.00–0.01) | 1.00 (1.00–1.00) | - | - |
|  | HRV | 0.59 (0.56–0.61) | 0.89 (0.88–0.90) | 0.54 (0.51–0.56) | 0.56 (0.54–0.58) |
|  | Influenza A | 0.00 (0.00–0.01) | 1.00 (1.00–1.00) | - | - |
|  | Influenza B | 0.72 (0.68–0.75) | 0.92 (0.92–0.93) | 0.42 (0.39–0.45) | 0.53 (0.50–0.56) |
|  | M.pneumoniae | 0.19 (0.17–0.22) | 0.98 (0.97–0.98) | 0.59 (0.54–0.64) | 0.29 (0.26–0.32) |
|  | RSV | 0.35 (0.31–0.38) | 0.98 (0.97–0.98) | 0.55 (0.50–0.60) | 0.43 (0.39–0.46) |
|  | SARS_CoV_2 | 0.88 (0.87–0.90) | 0.75 (0.74–0.76) | 0.50 (0.48–0.52) | 0.64 (0.62–0.65) |

RSV, respiratory syncytial virus; HCoV, human coronavirus; HPIV, human parainfluenza virus; HRV, human rhinovirus; SARS-CoV-2, severe acute respiratory syndrome coronavirus 2; HAdV, human adenovirus; M. pneumoniae, Mycoplasma pneumoniae.
